# RETRACTION: Diazepam Potentiates the Antidiabetic, Antistress and Anxiolytic Activities of Metformin in Type-2 Diabetes Mellitus with Cooccurring Stress in Experimental Animals

**DOI:** 10.1155/bmri/9821027

**Published:** 2025-08-07

**Authors:** BioMed Research International

RETRACTION: D. Garabadu and S. Krishnamurthy, “Diazepam Potentiates the Antidiabetic, Antistress and Anxiolytic Activities of Metformin in Type-2 Diabetes Mellitus with Cooccurring Stress in Experimental Animals,” *BioMed Research International* 2014 (2014): 693074, https://doi.org/10.1155/2014/693074

The above article, published online on 04 June 2014 in Wiley Online Library (http://wileyonlinelibrary.com), has been retracted by agreement between the Journal Section Editor, Jinsong Ren, and John Wiley & Sons Ltd. The retraction has been agreed following an investigation into concerns about the figures initially raised on PubPeer [1], specifically for Figures 3 and 4.

In Figure 3:

• In the p-IRS-1^ser307^ row, the DMS + MET+DZ band appears to be a mirror image of the control band, and the DMS + DZ band appears to be a mirror image of the DMS band.
• In the Total IRS row, the DMS + MET+DZ band appears to be a mirror image of the DMS band, and the DMS-DZ band appears to be a mirror image of the DMS + MET band.

In Figure 4:

• In the Total AKT row, the DMS + MET+DZ band appears to be a mirror image of the DMS band, and the DMS-DZ band appears to be a mirror image of the DMS + MET band.
• In the *β*-actin row, the Control and DMS bands appear to be duplicates.
• In the *β*-actin row, the DMS + MET+DZ band appears to be a mirror image of the DMS + DZ and DMS + MET bands.

The authors have responded to the concerns, however this was not deemed satisfactory by the editorial board, and retraction was recommended.

The authors did not respond when asked to agree to the retraction.
